# Highly Efficient Expression of Interleukin-2 under the Control of Rabbit β-Globin Intron II Gene Enhances Protective Immune Responses of Porcine Reproductive and Respiratory Syndrome (PRRS) DNA Vaccine in Pigs

**DOI:** 10.1371/journal.pone.0090326

**Published:** 2014-03-06

**Authors:** Yijun Du, Yu Lu, Xinglong Wang, Jing Qi, Jiyu Liu, Yue Hu, Feng Li, Jiaqiang Wu, Lihui Guo, Junzhen Liu, Haiying Tao, Wenbo Sun, Lei Chen, Xiaoyan Cong, Sufang Ren, Jianli Shi, Jun Li, Jinbao Wang, Baohua Huang, Renzhong Wan

**Affiliations:** 1 School of Life Sciences, Shandong University, Jinan, China; 2 Shandong Key Laboratory of Animal Disease Control and Breeding, Institute of Animal Science and Veterinary Medicine, Shandong Academy of Agricultural Sciences, Jinan, China; 3 National Research Center of Veterinary Biologicals Engineering and Technology, Jiangsu Academy of Agricultural Sciences, Nanjing, China; 4 College of Veterinary Medicine, Northwest A&F University, Yangling, China; 5 College of Animal Science & Veterinary Medicine, Shandong Agricultural University, Tai'an, China; 6 Department of Biology and Microbiology, Department of Veterinary and Biomedical Sciences, South Dakota State University, Brookings, South Dakota, United States of America; Virginia Polytechnic Institute and State University, United States of America

## Abstract

Highly pathogenic porcine reproductive and respiratory syndrome virus (HP-PRRSV) had caused catastrophic losses in swine industry in China. The current inactivated vaccine provided only limited protection, and the attenuated live vaccine could protect piglets against the HP-PRRSV but there was a possibility that the attenuated virus returned to high virulence. In this study, the eukaryotic expression vector pVAX1^©^ was modified under the control of rabbit β-globin intron II gene and the modified vector pMVAX1^©^ was constructed. Porcine interleukin-2 (IL-2) and GP3-GP5 fusion protein of HP-PRRSV strain SD-JN were highly expressed by pMVAX1^©^. Mice inoculated with pMVAX1^©^-GP35 developed significantly higher PRRSV-specific antibody responses and T cell proliferation than those vaccinated with pVAX1^©^-GP35. pMVAX1^©^-GP35 was selected as PRRS DNA vaccine candidate and co-administrated with pVAX1^©^-IL-2 or pMVAX1^©^-IL-2 in pigs. pMVAX1^©^-IL-2+pMVAX1^©^-GP35 could provide enhanced PRRSV-specific antibody responses, T cell proliferation, Th1-type and Th2-type cytokine responses and CTL responses than pMVAX1^©^-GP35 and pVAX1^©^-IL-2+pMVAX1^©^-GP35. Following homologous challenge with HP-PRRSV strain SD-JN, similar with attenuated PRRS vaccine group, pigs inoculated with pMVAX1^©^-IL-2+pMVAX1^©^-GP35 showed no clinical signs, almost no lung lesions and no viremia, as compared to those in pMVAX1^©^-GP35 and pVAX1^©^-IL-2+pMVAX1^©^-GP35 groups. It indicated that pMVAX1^©^-IL-2 effectively increases humoral and cell mediated immune responses of pMVAX1^©^-GP35. Co-administration of pMVAX1^©^-IL-2 and pMVAX1^©^-GP35 might be attractive candidate vaccines for preventing HP-PRRSV infections.

## Introduction

Porcine reproductive and respiratory syndrome virus (PRRSV) is a small, enveloped single-stranded, positive-sense RNA virus. It is a member of the genus *Arterivirus*, family *Arteriviridae*, order *Nidovirales* [1], [2]. The length of PRRSV genome is approximately 15 kb, and the genome codes for two large non-structural polyproteins (PP1a and PP1a/1b) in the 5′-terminal 12 kb region, and 8 structural proteins in the 3′-terminal 3 kb region: GP2 (glycoprotein 2), E (small envelope), GP3, GP4, GP5, GP5a, M (membrane), and N (nucleocapsid) proteins in order [3]–[5]. It was reported that immunization of GP3 and GP5 fusion protein has been shown to elicit enhanced immune responses [6] and GP3-GP5 has been selected as the candidate antigen of porcine reproductive and respiratory syndrome (PRRS) genetic engineering vaccines [7]–[10].

PRRS is one of the most economically significant viral diseases of swine, frustrating challenge to the global swine industry [11], [12]. In China, highly pathogenic PRRSV has been isolated and identified, causing a large-scale outbreak of PRRSV since 2006 [13]–[16]. Killed-PRRSV vaccines are less effective in prevention of infection [9]. Attenuated live vaccine could protect piglets from lethal challenge but there is a possibility that the attenuated virus returned to high virulence [17]. Thus, PRRS genetic engineering vaccines have been developed to control this disease [7], [18], [19]. However, we usually meet the cases that the DNA or recombinant protein cannot effectively induce immune response. An alternative approach is the co-delivery of cytokines to up-regulate the immune response [8], [9], [20], another one is using gene regulatory elements to improve the expression level of the protein antigen [21], [22].

IL-2 is an essential cytokine secreted mainly by activated T lymphocytes and plays a pivotal role in the replication and differentiation of T and B lymphocytes, monocytes, and natural killer cells [23]. IL-2 is one of cytokine adjuvants and enhances the protection against challenge with the infectious agent, including PRRSV [24]–[26]. However, the immune enhancing effects could not provide complete protection against PRRSV challenge [25], [26]. It might be related to the low expression level of IL-2 or using N protein of PRRSV as the antigen.

β-Globin Intron II gene was one of gene regulatory elements and used in expression systems to enhance the expression level of target proteins [21], [22]. It was found that addition of the rabbit β-Globin Intron II gene in DNA vaccine expressing the respiratory syncytial virus (RSV) fusion (F) protein (DNA-F) resulted in an increase of protein expression *in vitro* and critically contributed to the protection against respiratory syncytial virus infection *in vivo* [22]. The protein level expressed by commercially available pVAX1^©^ vector (Invitrogen, Carlsbad, CA, USA) was low. Whether insertion of the rabbit β-Globin Intron II gene to pVAX1^©^ vector could increase the protein expression level was never reported.

In this study, we first used the rabbit β-Globin Intron II gene as gene regulatory element to engineer the eukaryotic expression vector pVAX1^©^ and constructed the gene regulatory plasmid pMVAX1^©^. Then porcine IL-2 and GP3-GP5 of PRRSV were individually cloned into pVAX1^©^ and pMVAX1^©^. The immune responses of pMVAX1^©^-GP35 were evaluated in mice and the immune enhancing effects of pMVAX1^©^-IL-2 were examined in pigs. The results indicated that pMVAX1^©^-IL-2+pMVAX1^©^-GP35 is as effective as attenuated PRRS vaccine in pigs at protection against homologous HP-PRRSV challenge.

## Materials and Methods

### Ethics Statement

The animal experiments were approved by Jiangsu Provincial Science and Technology department in China and conducted accordingly. Experiments conformed to the local (Regulations for the administration of affairs concerning experimental animals) and international (Dolan K. 2007 Second Edition of Laboratory Animal Law. Blackwell, UK) guidelines on the ethical use of animals.

### Viruses and cells

HP-PRRSV strain SD-JN was kept in our laboratory [27]. MARC-145 cells were used to propagate and titrate HP-PRRSV SD-JN strain. The infected cell lysates were clarified, titrated, diluted to 1×10^5^ TCID_50_/ml and stored at −20°C to be used for animal challenge. The sixth passage (F6) MARC-145 cell culture supernatant was filtrated through a 0.45 µm filter and SD-JN PRRSV was concentrated from the supernatant by ultracentrifugation at 120,000×g (SW 40 rotor, Beckman) at 4°C for 2 h. The virus pellet was collected, diluted with TNE buffer [50 mM Tris-HCl (pH 7.5), 100 mM NaCl, 1 mM EDTA] and then layered on the top of 25–65% (w/v) sucrose gradients and at the same time centrifuged at 120,000×g (SW 40 rotor, Beckman) at 4°C for 4 h. The PRRSV particles band were harvested and pelleted at 120,000×g at 4°C for 2 h to remove the traces of sucrose. Then the PRRSV pellet was resuspended in phosphate buffered saline (PBS), quantitated by optical density (OD) measurement as described previously [6] and used for indirect ELISA (iELISA), T lymphocyte proliferation and cytokine assays. HEK-293A cells (ATCC CRL1573) were used for transfection of plasmids. All cells were grown in Dulbecco's modified Eagle's medium (DMEM) supplemented with 10% fetal bovine serum (FBS), 2 mM L-glutamine, 100 U penicillin/ml and 100 µg streptomycin/ml.

### RT-PCR for amplification of entire gene encoding for porcine IL-2

Based on porcine IL-2 gene sequence (GenBank accession no. NM_213861), one pair of PCR primers was designed as following: IL-2-Fwd: 5′-GAT*GAATTC*CACCATGGATAAGATGCAGC-3′ (containing *Eco*RI site italicized); IL-2-Rev: 5′-GCG*CTCGAG*TTAAGTCAGTGTTGAGTAGATGCTT-3′ (containing *Xho*I site italicized). Porcine spleen cells from 8-week-old Yorkshire swine were isolated by mechanical disruption and filtration through a 75 µm cell filter followed by hypotonic lysis of erythrocytes, then stimulated with ConA (10 µg/ml; Sigma-Aldrich, St. Louis, MO, USA) for 24 h *in vitro*. Total RNA was extracted using TRIzol reagent (Invitrogen, Carlsbad, CA, USA) as the manufacturer's protocol. The cDNA was synthesized using oligo d(T)_12-18_ primer. Then the entire gene encoding for porcine IL-2 (487 bp) was amplified from the cDNA. The amplification was performed in a 50 µl reaction mixture containing 1.5 mM MgCl_2_, 1×PCR buffer, 0.2 mM of each dNTP, 20 pmol of each primer, 1.5 U of TaqDNA polymerase (Invitrogen, Carlsbad, CA, USA) and 2 µl of the cDNA. The reaction was run in a thermocycler (DNA Engine, PTC-0200; Bio-Rad Laboratories, Hercules, CA, USA) with the following program: denaturation at 94°C for 5 min, 30 cycles composed of denaturation at 94°C for 40 s, annealing at 60°C for 40 s and extension at 72°C for 1 min, and was ended with a final extension step of 10 min at 72°C.

### Modification of the pVAX1^©^ vector

**Figure 1:**
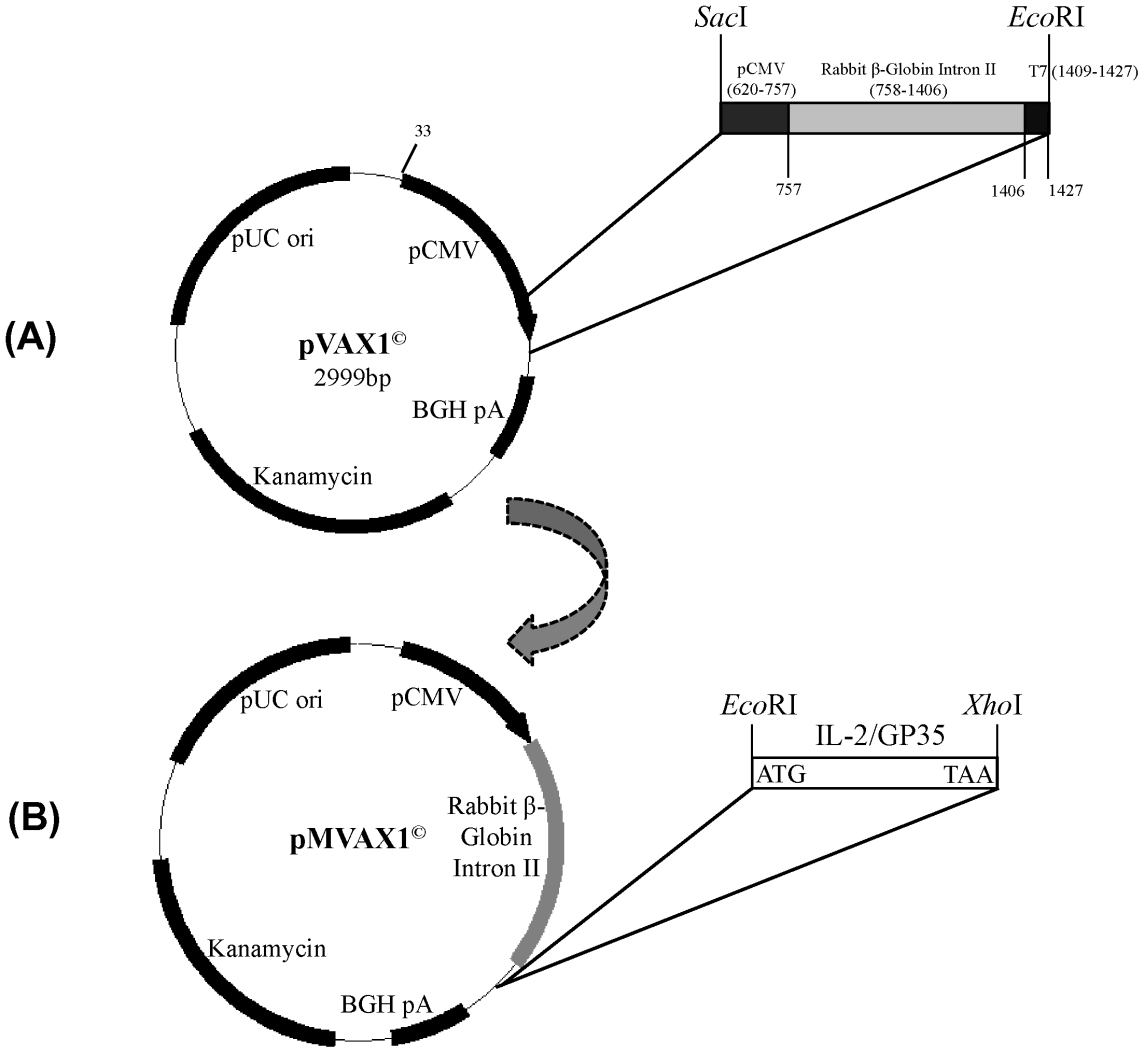
Schematic construction of gene regulatory plasmid pMVAX1^©^ and recombinant plasmids. (A) The partial CMV promoter (138 bp, position 620-757), rabbit β-Globin Intron II (649 bp, position 758-1406) and T7 promoter gene (19 bp, position 1409-1427) were cloned into pVAX1^©^ vector, and pMVAX1^©^ was constructed. (B) The IL-2 and GP3-GP5 gene were inserted into pMVAX1^©^ vector, respectively.

Based on the rabbit β-Globin Intron II gene regulatory sequence (GenBank accession no. V00882), the partial CMV promoter (138 bp, position 620-757), rabbit β-Globin Intron II (649 bp, position 758-1406) and T7 promoter gene (19 bp, position 1409-1427) were fused and chemically synthesized by Invitrogen Biotechnology Co. Ltd. (Shanghai, China), with *Sac*I and *Eco*RI flanking the both sides. The whole gene was digested with *Sac*I and *Eco*RI and cloned into pVAX1^©^ vector (Invitrogen, Carlsbad, CA, USA) (Fig. 1A). The eukaryotic expression vector pVAX1^©^ was modified and the gene regulatory plasmid pMVAX1^©^ was constructed (Fig. 1A).

### Construction of the expression plasmids pVAX1^©^-IL-2, pMVAX1^©^-IL-2 and pMVAX1^©^-GP35

The PCR product of IL-2 gene was digested with *Eco*R I and *Xho* I and inserted into pMVAX1^©^ or pVAX1^©^ vector to produce pMVAX1^©^-IL-2 and pVAX1^©^-IL-2 (Figs. 1A and 1B).

The construction of pVAX1^©^-GP35 expressing GP3-GP5 was described elsewhere and GP3-GP5 was expressed as a fusion protein [10]. In order to generate pMVAX1^©^-GP35 expressing GP3-GP5, GP3-GP5 gene was amplified from plasmid pVAX1^©^-GP35 using primer pair as following: GP3-1 (upstream primer): 5′-TAT*GAATTC*CACCATGGCTAATAGCTGTACA-3′ (containing *Eco*RI site italicized); GP5-Rev (downstream primer): 5′-GAG*CTCGAG*TTACTAGAGACGACCCC-3′ (containing *Xho*I site italicized). Then the GP3-GP5 gene was cloned into pMVAX1^©^ vector using *Eco*RI and *Xho*I sites (Fig. 1B).

All the expression plasmids were sequenced to confirm the correct tandem in frame insertion of individual gene.

### Transfection

HEK-293A cells were seeded in 35-mm diameter dishes and grown to 70% confluency. Cells were transfected with 2.0 µg of empty vector pVAX1^©^, pMVAX1^©^, pVAX1^©^-IL-2, pMVAX1^©^-IL-2, pVAX1^©^-GP35 or pMVAX1^©^-GP35 using Lipofectamine™ 2000 according to the manufacturer's instructions (Invitrogen, Carlsbad, CA, USA). Cells were transfected with individual plasmid in duplicate. At 24 h post-transfection, cells of one dish were washed with PBS and lysed in cell lysis buffer supplemented with PMSF (Beyotime, China) for western blotting analysis. Cells of the other dish were frozen and thawed three times for detecting the bioactivity of IL-2.

### Western blotting assay

Western blotting was carried out as described previously [10]. Briefly, the cell lysates were resolved in a 10% polyacrylamide gel. The separated proteins were then transferred onto polyvinylidene difluoride (PVDF) membranes (Millipore, Billerica, MA, USA) and probed with PRRSV-specific antiserum of pigs or mouse monoclonal antibody against swine IL-2 (Invitrogen, Camarillo, CA, USA), respectively. Specific reaction products were detected with horseradish peroxidase-conjugated Staphylococcal Protein A (SPA-HRP, Boshide, Wuhan, China) or HRP-conjugated goat anti-mouse IgG (Boshide, Wuhan, China). The membranes were developed using Supersignal West Pico Chemiluminescent Substrate according to the manufacturer's suggestions (Pierce, Rockford, IL, USA). Digital signal acquisition and analysis were conducted by the Quantity One program, version 4.6 (Bio-Rad).

### Bioactivity of IL-2 expressed by pVAX1^©^-IL-2 and pMVAX1^©^-IL-2

Cells transfected with individual plasmid were frozen and thawed three times and then centrifuged at 13,000 rpm for 10 min in a microcentrifuge (Eppendorf 5415R). Supernatants were harvested and the bioactivity of IL-2 was examined as described before [28], with a little modification. Briefly, porcine spleen from 8-week-old Yorkshire swine was collected aseptically in PBS. Part of the spleen (about 50 g) was cut, minced with scissors and then passed through a 75 µm cell filter to obtain a homogeneous cell suspension. The spleen cells were collected after centrifugation at 250×g for 10 min at 4°C and resuspended in Hank's balanced salt solution (HBSS). Cell suspension was then overlaid on equal volume of Histopaque-1077 (Sigma-Aldrich, St. Louis, MO, USA) and centrifuged at 1,000×g. Cells were washed twice in serum-free RPMI-1640 and planted in six well cell culture plates in RPMI-1640 medium containing 10% FBS at a concentration of 1×10^7^ cells/ml. The cells were stimulated with ConA (10 µg/ml; Sigma-Aldrich) for 24 h and the residual ConA in the cell supernatant was removed by incubating with 0.1 M α-methyl D-mannoside for 30 min. The endogenous IL-2 protein in the supernatant was purified according to the previous report [28]. The stimulated cells were applied to Histopaque-1077 (Sigma-Aldrich), and live cells were selected after centrifugation. The enriched viable cells were resuspended in RPMI-1640 medium containing 10% FBS and cultured at 5×10^6^ cells/ml with two-folds dilutions of the supernatants from cells transfected with individual plasmid in triplicates in 96-well plates (100 µl per well). RPMI-1640-FBS only was used as negative control. The purified endogenous IL-2 protein was used as positive control. After incubation at 37°C for 48 h, 20 µl MTT (3-(4, 5-dimethylthiazol-2-yl) 2, 5-diphenyltetrazolium bromide, 5 mg/ml) was added to each well and reacted for 4.5 h. Then 100 µl of lysis buffer (20% SDS/50% DMF) was added. Plates were incubated for 20 h at 37°C and the OD values were measured at 570 nm. OD _IL-2_/OD _negative_ ≥1.5 was defined as the positive criteria for the biological activity of IL-2.

### Preparation of DNA plasmids

All plasmids for DNA immunizations were grown in *E.coli* DH5α strain (Invitrogen, Carlsbad, CA, USA), and large-scale preparation of the plasmid DNA was carried out using Qiagen EndoFree Plasmid-Giga kits (Qiagen, Chatsworth, CA, USA) according to the manufacturer's instructions.

### Animal experiments

The ARRIVE Guidelines Checklist-NC3Rs for Animal Research was provided in Table S1.

#### Immunization of mice

A total of 75, 6-week-old female BALB/c mice (provided by the Animal Center of Nanjing Army Hospital, Nanjing, China) were randomly divided into 5 groups each with 15. Groups 1–4 were individually inoculated with 100 µg of pVAX1^©^, pMVAX1^©^, pVAX1^©^-GP35 and pMVAX1^©^-GP35 in 0.2 ml PBS. Group 5 was inoculated with 0.2 ml PBS. All groups of mice were injected intradermally twice at 3-week intervals using regular syringes and needles. At 21, 35 and 49 days post primary immunization (dpi), five mice from each group were euthanized and the sera were harvested for the detection of antibodies against PRRSV using iELISA and serum neutralization (SN) assays. The lymphocytes were separated from the spleen of each mouse at 35 and 49 dpi for the detection of PRRSV-specific cell mediate immune responses.

#### Vaccination of pigs

Forty-five 21-day-old crossbreed (Landrace×local stock) pigs were obtained from a local farm without PRRSV, porcine circovirus 2 (PCV-2), porcine parvovirus (PPV), pseudorabies virus (PRV) and Actinobacillus pleuropneumoniae (APP) history. All pigs were tested and proven to be seronegative for PRRS by iELISA and PRRSV negative by RT-PCR. The animals were then randomly divided into 9 groups, numbered, and housed in separate rooms. Group 1 was injected with 1 ml PBS. Groups 2–6 were individually injected with 500 µg of pVAX1^©^, pMVAX1^©^, pVAX1^©^-IL-2, pMVAX1^©^-IL-2 and pMVAX1^©^-GP35 in 1 ml PBS. Groups 7 and 8 were inoculated with pVAX1^©^-IL-2 (500 µg)+pMVAX1^©^-GP35 (500 µg) and pMVAX1^©^-IL-2 (500 µg)+pMVAX1^©^-GP35 (500 µg) in 1 ml PBS, respectively. Group 9 was vaccinated with commercial HP-PRRS live vaccine (1×10^5^ TCID_50_ in 1 ml PBS, Attenuated PRRS vaccine, Strain JXA1-R, Guangdong Dahuanong Animal Health Products Co., Ltd, China). The plasmid DNA, attenuated PRRS vaccine or PBS was injected in the cervical region muscles using regular syringes and needles and the immunization was boosted 28 days later. The sera were collected from each pig at 28, 42 and 56 dpi to detect antibodies to PRRSV using iELISA and SN assays. At 42 and 56 dpi, the heparinized blood was used to isolate peripheral blood mononuclear cells (PBMCs) for T lymphocyte proliferation assay. At 42 dpi, PBMCs were isolated from the blood of pigs and stimulated with purified SD-JN PRRSV antigen (10 µg/ml). The supernatant was obtained to detect the levels of Th1-type cytokine IFN-γ and Th2-type cytokine IL-4. PBMCs isolated from pigs at 42 dpi were also used for Cytotoxic T-lymphocyte (CTL) assay. At 56 dpi, all pigs were challenged intramuscularly with 1×10^5^ TCID_50_ PRRSV SD-JN strain (F6 passage, 1 ml) using regular syringes and needles. And then the animals were monitored daily for rectal temperatures and clinical signs until 21 days post challenge (dpc).

#### iELISA

The purified SD-JN PRRSV antigen was used as iELISA antigen and coated in 96-well plates at the concentration of 1.0 µg/ml. The plates were blocked with 0.15% BSA in PBS. The sera of mice or pigs were diluted 1∶2 serially in PBS-T (PBS containing 0.5% Tween80, PBS-T) and added into the plates. 3 wells were repeated per dilution. After incubation for 60 min at 37°C, the wells were washed with PBS-T for three times and incubated with Goat anti-mouse IgG-HRP or SPA-HRP (Boshide, Wuhan, China) for 60 min at 37°C. The plates were incubated with substrate solution O-phenilendiamine (OPD) at 37°C for 15 min and the reaction was stopped by adding 2 M H_2_SO_4_ solution in each well. The OD values were read at 490 nm in an ELISA reader. Meanwhile, the PRRSV negative sera of mice or pigs were used as negative controls. The results were expressed as the ratio of OD_490 nm_ produced by the serum samples compared to negative control serum. Sera, giving a ratio value higher than 2.1, were considered to be positive sera. The titers were expressed as the highest dilution of antibody producing 2.1 ratio value [6], [10].

#### SN assays

SN assays were performed as previously described [6]. All serum samples from mice and pigs were heat inactivated (56°C, 30 min) and 1∶2 serially diluted. Then, the serial dilutions of serum were mixed with equal volumes of 200 TCID_50_ SD-JN PRRSV. After incubation at 37°C for 1 h, the mixtures were transferred to MARC-145 monolayers in 96-well tissue culture plates. Then, the plates were incubated and observed daily for up to 5 days for the appearance of CPE. Meanwhile, the PRRSV positive and negative sera of pigs were used as positive and negative controls, respectively. CPE was used to determine the end-point titers that were calculated as the reciprocal of the last serum dilution to neutralize 100 TCID_50_ of PRRSV in 50% of the wells.

#### T lymphocyte proliferation assay

Lymphocytes were isolated individually from the spleen of each mouse or heparinized blood of pigs with lymphocyte separation medium (Boshide, Wuhan, China), suspended to 5×10^6^/ml with RPMI complete medium (RPMI-1640 containing 10% FBS, 2 mM L-glutamine, 50 µM β-mercaptoethanol, 200 U/ml penicillin, 200 µg/ml streptomycin, 100 U/ml of mycostatin) and seeded in 96-well flat-bottom plates at 100 µl per well. Each cell sample was plated in triplicate. The culture was stimulated with purified SD-JN PRRSV antigen at a final concentration of 10 µg/ml or unstimulated, respectively. Meanwhile, phytohemagglutinin (PHA) (10 µg/ml; Sigma-Aldrich) was used as a positive control. After incubation for 45 h at 37°C with 5% CO_2_, the proliferation responses were detected by a standard MTT method. T lymphocyte proliferation was expressed as stimulation index (SI), which is the ratio of OD_570 nm_ of stimulated well to that of unstimulated one [6], [10].

#### Cytokine assays

Subsets of Th cells can be distinguished by their pattern of cytokine expression. Th1 cells produce IFN-γ, IL-2, and lymphotoxin, and Th2 cells produce IL-4, IL-5, IL-10 and IL-13. To distinguish the subsets, PBMCs (5×10^6^/ml, 100 µl per well) isolated from the blood of pigs were stimulated with purified SD-JN PRRSV antigen at the final concentration of 10 µg/ml. After 72 h, the cells were centrifuged and the supernatant was collected to examine the levels of the Th1-type cytokine IFN-γ and Th2-type cytokine IL-4 using commercially available porcine IFN-γ/IL-4 ELISA kits (Biosource, Camarillo, CA, USA) following the manufacturer's instructions.

#### CTL assay

CTL assay was carried out as described previously [29], with a little modification. Briefly, 21-day-old pig free of PRRSV, tested to be seronegative for PRRS by iELISA and SN assays, was used for collecting PAMs by lung lavage. PAMs were incubated with PRRSV SD-JN at an MOI of 0.01 for 24 h and used as the target cells. PBMCs isolated from pigs at 42 dpi were used as effector cells. 1×10^7^/ml and 5×10^6^/ml of effector cells were suspended in phenol red-free RPMI-1640 medium containing 2% BSA, 2 mM glutamine, and 1% penicillin and streptomycin, and seeded in 96-well round-bottom plates with 100 µl per well. Each cell sample was plated in triplicate. Subsequently 100 µl of the target cells (1×10^4^/well) was added in each well and incubated for 4–6 h at 37°C with 5% CO_2_. The supernatant was harvested, and the substrate tetrazolium salt was added. The OD values were read at 490 nm in an ELISA reader. The specific lactate dehydrogenase (LDH) activity release was calculated by using following formula: (experimental release-spontaneous release)/(maximum release-spontaneous release) ×100.

#### Clinical evaluations and gross lesions

The severity of the clinical signs was evaluated daily after challenge as reported [8], [10]. Observations included behavior, respiration and cough. Scores for each of three individual observations ranged from 1 to 4. The overall score for clinical condition was determined by sum of daily observations of behavior, respiration and cough. For example, a clinically normal animal would be given a total score of 3 (i.e., behavior = 1, respiration = 1, cough = 1), an animal with maximum clinical illness would be given a total score of 9 (i.e., behavior = 3, respiration = 3, cough = 3) and a dead animal would be given a total score of 12 (i.e., behavior = 4, respiration = 4, cough = 4). Sequential blood samples were collected from all animals at 0, 7, 14 and 21 dpc for isolation of PRRSV. The experiments were terminated on 21 dpc and all the animals were humanely euthanized. The gross lesions of lungs were evaluated at necropsy. Lungs were evaluated by the percentage of lesions noted per lobe, and then, using a standard scoring system, an overall level of gross lung pathology was determined [30]. The histological pathology of lungs was determined as described [31].

#### Viremia assessment

Viremia in challenged pigs was determined as previously described [32]. MARC-145 cells were seeded in 12-well plates and grown to 70–80% confluence. Cells were inoculated with respective serum samples for 1 h and DMEM supplemented with 2% FBS, 2 mM L-glutamine, 100 U penicillin/ml and 100 µg streptomycin/ml was added. CPE in each MARC-145 cell culture well was observed microscopically for 5 days and the percentage of wells positive for CPE was calculated per group.

### Statistical analysis

Data were compared and the differences were determined by One-way repeated measurement ANOVA and Least significance difference (LSD). A *P*-value <0.05 was considered statistically significant [8].

## Results

### Construction of the expression plasmids pVAX1^©^-IL-2, pMVAX1^©^-IL-2 and pMVAX1^©^-GP35

The partial CMV promoter, rabbit β-Globin Intron II and T7 promoter gene were successfully cloned into pVAX1^©^ vector and the gene regulatory plasmid pMVAX1^©^ was obtained (Fig. 1A). The whole sequence of pMVAX1^©^ was deposited in the GenBank (Accession no. KF703899). DNA sequencing confirmed that the nucleotide sequence of the insert genes in recombinant plasmids pVAX1^©^-IL-2, pMVAX1^©^-IL-2 and pMVAX1^©^-GP35 had the same sequence as designed, as well as in the proper open reading frame.

### Expression analysis of pVAX1^©^-IL-2, pMVAX1^©^-IL-2 and pMVAX1^©^-GP35 in HEK-293A cells

**Figure 2:**
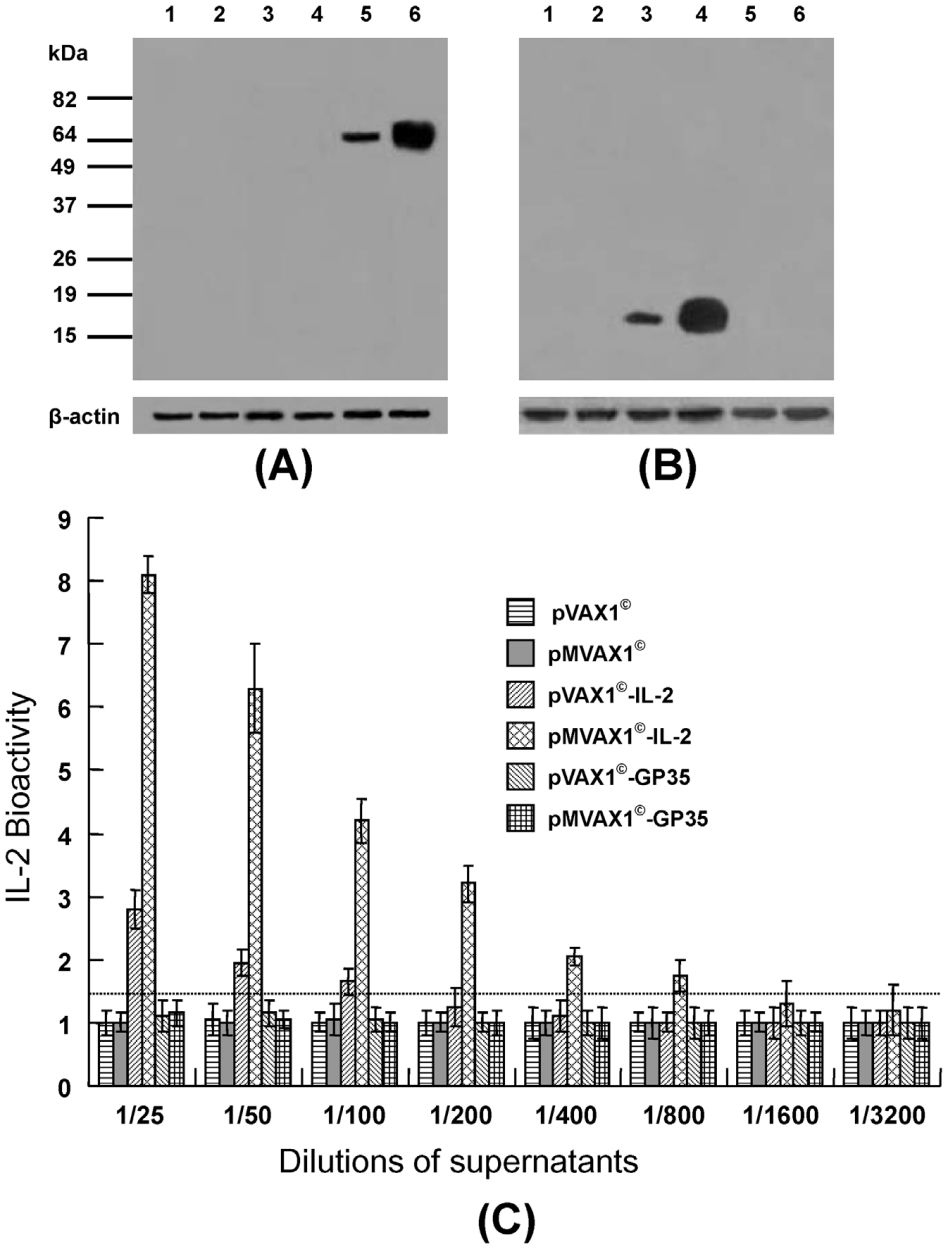
Western blotting analysis of total lysates of HEK-293A cells transfected with pVAX1^©^ (lane 1), pMVAX1^©^ (lane 2), pVAX1^©^-IL-2 (lane 3), pMVAX1^©^-IL-2 (lane 4), pVAX1^©^-GP35 (lane 5) or pMVAX1^©^-GP35 (lane 6), respectively, using anti-PRRSV serum of pigs (A) or mouse monoclonal antibody against swine IL-2 (B). (C) Bioactivity examination of IL-2 in the supernatants from cells transfected with individual plasmid. The negative control of RPMI-1640-FBS showed a stimulation index of 1–1.2. The positive control of the purified endogenous IL-2 protein showed a stimulation index of 4–6.

The expressions of GP35 and IL-2 were examined by western blotting with PRRSV-specific antiserum of pigs or monoclonal antibody against swine IL-2. As shown in Fig. 2A, the specific band of ∼65–70 kDa, being consistent with the predicted size of GP35, was clearly observed in cell lysates of pVAX1^©^-GP35 and pMVAX1^©^-GP35 as visualized with PRRSV-specific antiserum of pigs, whereas no band was found in cell lysates of pVAX1^©^, pMVAX1^©^, pVAX1^©^-IL-2 or pMVAX1^©^-IL-2. In addition, the expression level of pMVAX1^©^-GP35 was 4.3-fold higher than that of pVAX1^©^-GP35, as analyzed by the Quantity One program and normalized with β-actin. HEK-293A cells transfected with pVAX1^©^-IL-2 or pMVAX1^©^-IL-2 produced the specific band of IL-2 (17 kDa) and the expression level of pMVAX1^©^-IL-2 was 5.7-fold higher than that of pVAX1^©^-IL-2 (Fig. 2B).

To further detect the expression of IL-2, the bioactivity of IL-2 was examined. The activated swine spleen cells were co-cultured with the serial two-folds dilutions of the supernatants from cells transfected with individual plasmid. Then the culture mixtures reacted for 4.5 h with MTT. Data in Fig. 2C showed that 1/100 dilution of the supernatant from cells transfected with pVAX1^©^-IL-2 significantly stimulated the splenic lymphocyte proliferation, while for pMVAX1^©^-IL-2, only 1/800 dilution was needed. In addition, IL-2 expressed by pVAX1^©^-IL-2 and pMVAX1^©^-IL-2 could stimulate the lymphocyte proliferation in a dose-dependent manner. The positive control of the purified endogenous IL-2 protein showed a stimulation index of 4–6 (data not shown). In contrast, RPMI-1640-FBS only, the supernatants from cells transfected with pVAX1^©^, pMVAX1^©^, pVAX1^©^-GP35 and pMVAX1^©^-GP35 showed no stimulation. These results indicated that the bioactivity of IL-2 expressed by pMVAX1^©^-IL-2 was significant higher than that of pVAX1^©^-IL-2.

### Humoral immune responses of pVAX1^©^-GP35 and pMVAX1^©^-GP35 in mice

**Figure 3:**
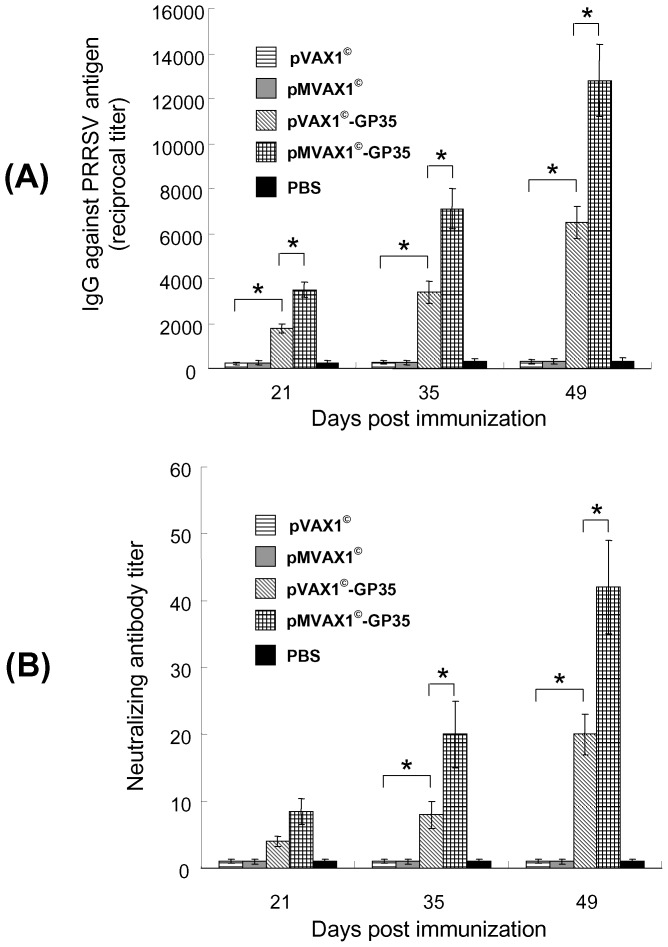
PRRSV-specific antibody responses in mice immunized with PBS, pVAX1^©^, pMVAX1^©^, pVAX1^©^-GP35 or pMVAX1^©^-GP35. (A) iELISA analysis of sera from mice immunized with PBS, pVAX1^©^, pMVAX1^©^, pVAX1^©^-GP35 or pMVAX1^©^-GP35. Serum samples (n = 5) were collected at various time-points and antibodies to purified SD-JN PRRSV antigen were detected using iELISA. The sera of mice were diluted 1∶2 serially in PBS-T and added into the plates. 3 wells were repeated per dilution. Then the experiment was performed according to the M&M. The results were expressed as the highest dilution of antibody producing 2.1 ratio value. Data were shown as mean±standard error. (B) Neutralizing antibody responses in mice immunized with PBS, pVAX1^©^, pMVAX1^©^, pVAX1^©^-GP35 or pMVAX1^©^-GP35. Serum samples (n = 5) were collected at various time-points and detected by SN assays. The titers of neutralizing antibodies were expressed as the reciprocal of the last serum dilution to neutralize 100 TCID_50_ of PRRSV in 50% of the wells [6], [10]. Data were shown as mean±standard error.

The sera collected from mice at 21, 35 and 49 dpi were used to detect the PRRSV-specific antibody levels. As shown in Fig. 3A, anti-PRRSV antibodies in mice vaccinated with pVAX1^©^-GP35 and pMVAX1^©^-GP35 could be detected by ELISA at 21 dpi, and markedly increased after the booster. pMVAX1^©^-GP35 group induced significantly higher levels of PRRSV-specific antibodies at 21, 35 and 49 dpi comparing with pVAX1^©^-GP35 group (*P*<0.05).

Sera samples were further evaluated the ability to neutralize PRRSV *in vitro* by SN assays. The results indicated that the levels of neutralizing antibodies in pMVAX1^©^-GP35 group were also markedly higher than those in pVAX1^©^-GP35 group at 35 and 49 dpi (Fig. 3B). No neutralizing antibodies against PRRSV could be detected in mice immunized with pVAX1^©^, pMVAX1^©^ or PBS.

### T lymphocyte proliferation responses of pVAX1^©^-GP35 and pMVAX1^©^-GP35 in mice

**Figure 4:**
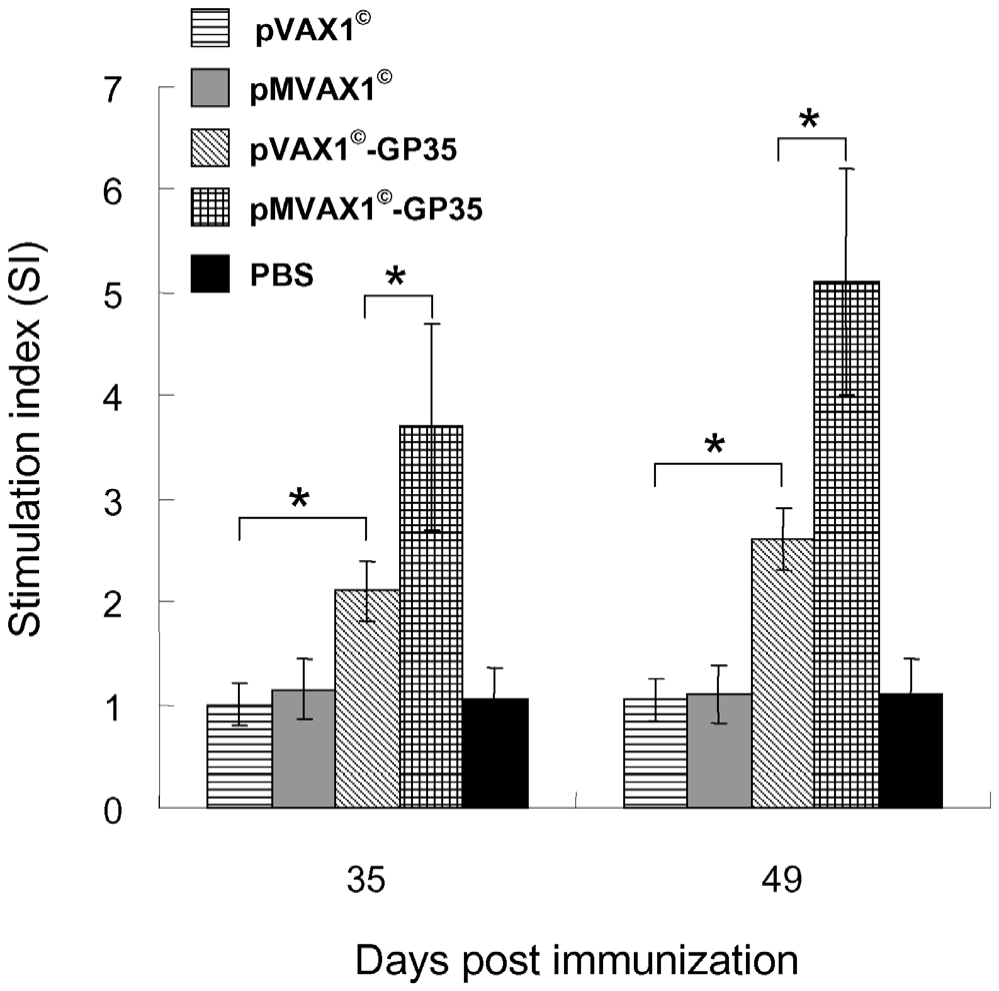
Lymphocyte proliferative responses in mice immunized with PBS, pVAX1^©^, pMVAX1^©^, pVAX1^©^-GP35 or pMVAX1^©^-GP35. Splenocytes samples (n = 5) were collected at days 35 and 49 dpi and were stimulated with purified SD-JN PRRSV antigen (10 µg/ml) in triplicate. After 45 h of stimulation, MTT was added and the proliferation responses were detected by a standard MTT method [6], [10]. The PHA control sample showed a stimulation index of 6–8. Data were shown as mean±standard error.

At days 35 and 49 dpi, spleen cells were pooled and the PRRSV-specific T lymphocyte proliferation responses were detected. The results showed that the pMVAX1^©^-GP35 could induce significantly higher levels of the proliferation at 35 and 49 dpi, comparing with pVAX1^©^-GP35 (*P*<0.05) (Fig. 4).

As pMVAX1^©^-GP35 could induce significantly higher PRRSV-specific humoral immune responses and cell mediated immune responses than pVAX1^©^-GP35, pMVAX1^©^-GP35 was selected as PRRS DNA vaccine candidate and further studied in pigs.

### Enhanced humoral immune responses induced by pMVAX1^©^-IL-2+pMVAX1^©^-GP35 in pigs

**Figure 5:**
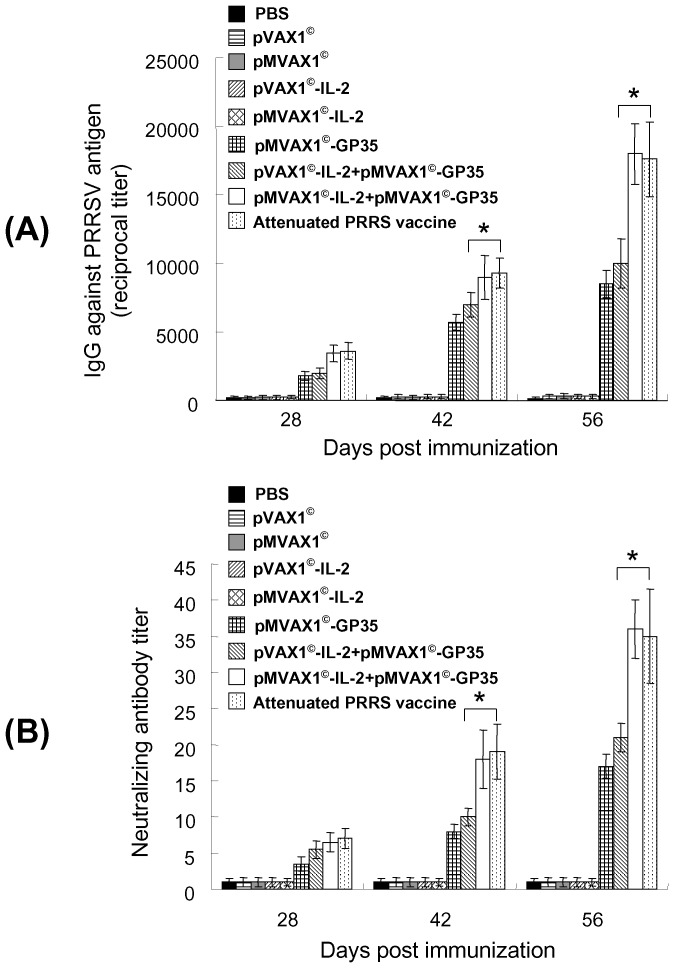
PRRSV-specific antibody responses in pigs immunized with PBS, individual plasmid or attenuated PRRS vaccine. (A) iELISA analysis of sera from pigs immunized with PBS, individual plasmid or attenuated PRRS vaccine in the cervical region muscles using regular syringes and needles. Serum samples (n = 5) were collected at various time-points and antibodies to purified SD-JN PRRSV antigen were detected using iELISA. Data were shown as mean±standard error. (B) Neutralizing antibody responses in pigs immunized with PBS, individual plasmid or attenuated PRRS vaccine in the cervical region muscles using regular syringes and needles. Serum samples (n = 5) were collected at various time-points and detected by SN assays. The titers of neutralizing antibodies were expressed as the reciprocal of the last serum dilution to neutralize 100 TCID_50_ of PRRSV in 50% of the wells [6], [10]. Data were shown as mean±standard error.

The immunogenicity of the proteins expressed from these plasmids was further investigated in pigs. The serum took at 28, 42 and 56 dpi were used to detect the PRRSV-specific antibody levels. As shown in Fig. 5A, the levels of IgG from the groups of pMVAX1^©^-IL-2+pMVAX1^©^-GP35 and attenuated PRRS vaccine were significantly higher than those from the group of pMVAX1^©^-GP35 at 42 and 56 dpi (*P*<0.05). Moreover, the levels of IgG from the groups of pMVAX1^©^-IL-2+ pMVAX1^©^-GP35 and attenuated PRRS vaccine were also significantly higher than the group that received pVAX1^©^-IL-2+pMVAX1^©^-GP35 (*P*<0.05). There was no significant difference between the group of pMVAX1^©^-IL-2+pMVAX1^©^-GP35 and attenuated PRRS vaccine (*P*>0.05).

The results of SN assays indicated that pMVAX1^©^-IL-2+pMVAX1^©^-GP35 group provided significantly higher levels of neutralizing antibodies than pVAX1^©^-IL-2+pMVAX1^©^-GP35 and pMVAX1^©^-GP35 groups (*P*<0.05). pMVAX1^©^-IL-2 +pMVAX1^©^-GP35 could induce similar neutralizing antibodies with attenuated PRRS vaccine (*P*>0.05). Meanwhile, no neutralizing antibodies was detected in PBS, pVAX1^©^, pMVAX1^©^, pVAX1^©^-IL-2 and pMVAX1^©^-IL-2 groups (Fig. 5B). These results indicated that pMVAX1^©^-IL-2 could significantly enhance the humoral immune responses of PRRS DNA vaccine pMVAX1^©^-GP35.

### Enhanced T lymphocyte proliferation responses induced by pMVAX1^©^-IL-2+pMVAX1^©^-GP35 in pigs

**Figure 6:**
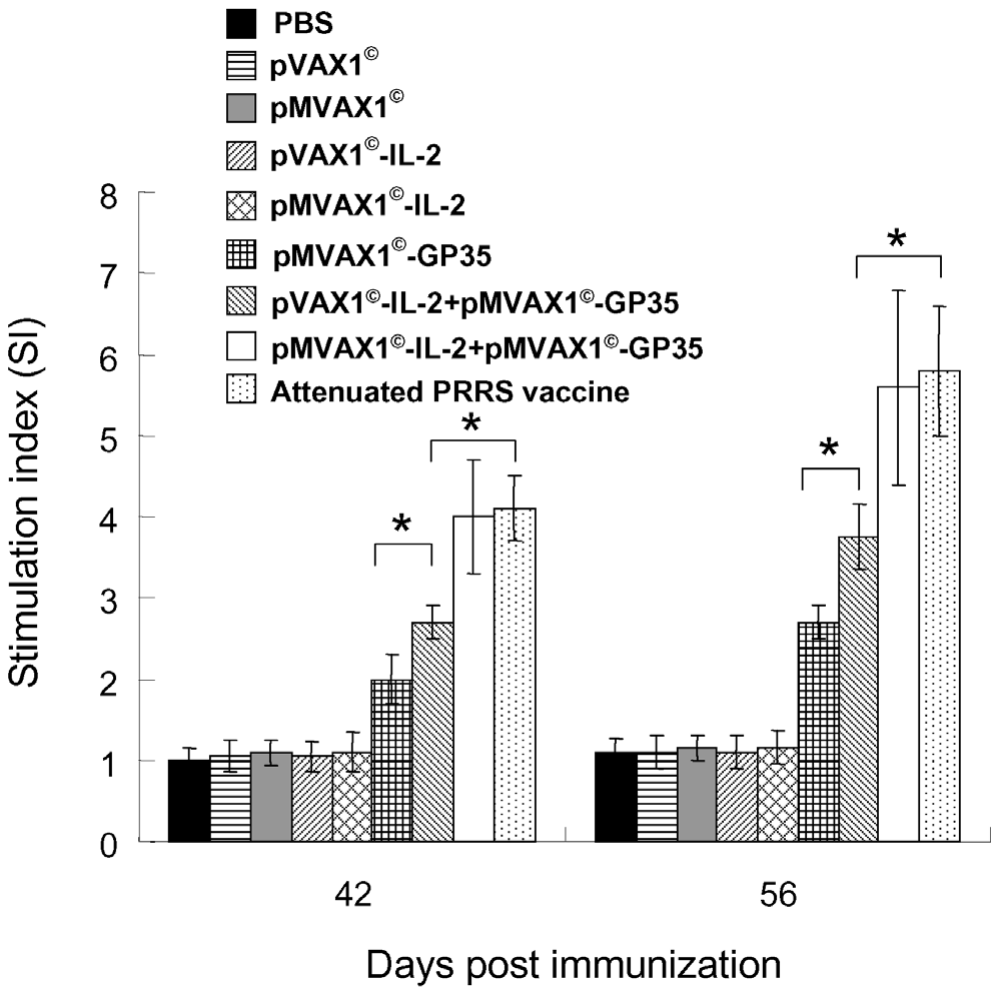
Lymphocyte proliferative responses in pigs immunized with PBS, individual plasmid or attenuated PRRS vaccine in the cervical region muscles using regular syringes and needles. Heparinized blood samples (n = 5) were collected at days 42 and 56 dpi and PBMCs were stimulated with purified SD-JN PRRSV antigen (10 µg/ml) in triplicate. After 45 h of stimulation, MTT was added and the proliferation responses were detected by a standard MTT method [6], [10]. The PHA control sample showed a stimulation index of 6–8. Data were shown as mean±standard error.

As shown in Fig. 6, PRRSV-specific lymphocyte proliferation responses from the group of pMVAX1^©^-IL-2+pMVAX1^©^-GP35 were significantly higher than those from the group of pVAX1^©^-IL-2+pMVAX1^©^-GP35 at 42 and 56 dpi (*P*<0.05). Moreover, the responses from the group of pVAX1^©^-IL-2+pMVAX1^©^-GP35 were also significantly higher than the group that received pMVAX1^©^-GP35 (*P*<0.05). There was no significant difference between the group of pMVAX1^©^-IL-2+pMVAX1^©^-GP35 and attenuated PRRS vaccine (*P*>0.05). Pigs inoculated with PBS, pVAX1^©^, pMVAX1^©^, pVAX1^©^-IL-2 or pMVAX1^©^-IL-2 could not induce PRRSV-specific T lymphocyte proliferation responses. This result revealed that pMVAX1^©^-IL-2 significantly improved the cell mediated immune responses of pMVAX1^©^-GP35.

### Enhanced cytokine responses induced by pMVAX1^©^-IL-2+pMVAX1^©^-GP35 in pigs

**Figure 7:**
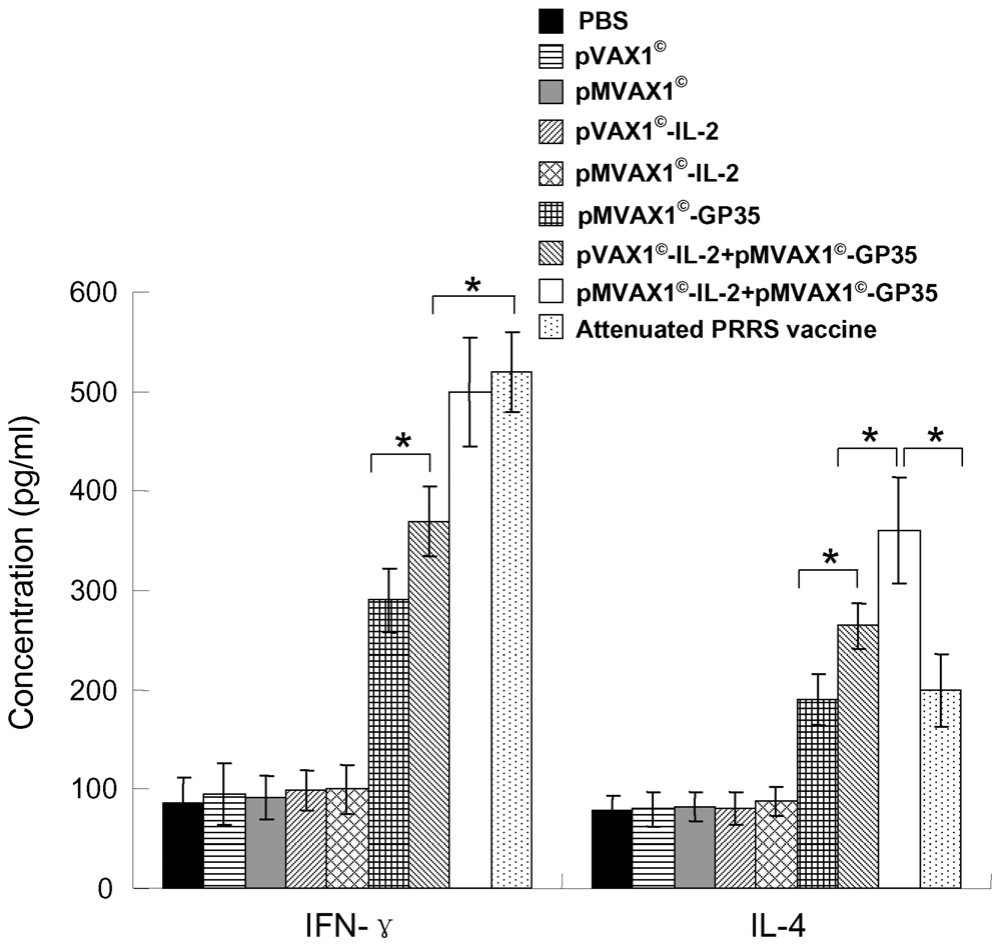
The concentrations (pg/ml) of Th1-type cytokine IFN-γ and Th2-type cytokine IL-4 in the supernatants. PBMCs (5×10^6^/ml, 100 µl per well) were isolated from the blood of pigs at 42 dpi and stimulated with purified SD-JN PRRSV antigen (10 µg/ml). After 72-type cytokine IFN-γ and Th2-type cytokine IL-4 using commercially available pig cytokine ELISA kits. Data were shown as mean±standard error.

Swine cytokines detection kits were employed to detect the production of IFN-γ and IL-4 in the supernatant of the lymphocytes stimulated with purified SD-JN PRRSV antigen at 42 dpi. The results showed that the levels of IFN-γ and IL-4 were significant higher in group that received pMVAX1^©^-IL-2+pMVAX1^©^-GP35, comparing with those of pMVAX1^©^-GP35 and pVAX1^©^-IL-2+pMVAX1^©^-GP35 (*P*<0.05, Fig. 7). Attenuated PRRS vaccine could induce similar high level of IFN-γ with pMVAX1^©^-IL-2+pMVAX1^©^-GP35, but the level of IL-4 was lower (*P*<0.05). The results demonstrated that pMVAX1^©^-IL-2+pMVAX1^©^-GP35 could potentiate both Th1-type and Th2-type cytokine responses.

### Enhanced CTL induction by pMVAX1^©^-IL-2+pMVAX1^©^-GP35 in pigs

**Figure 8:**
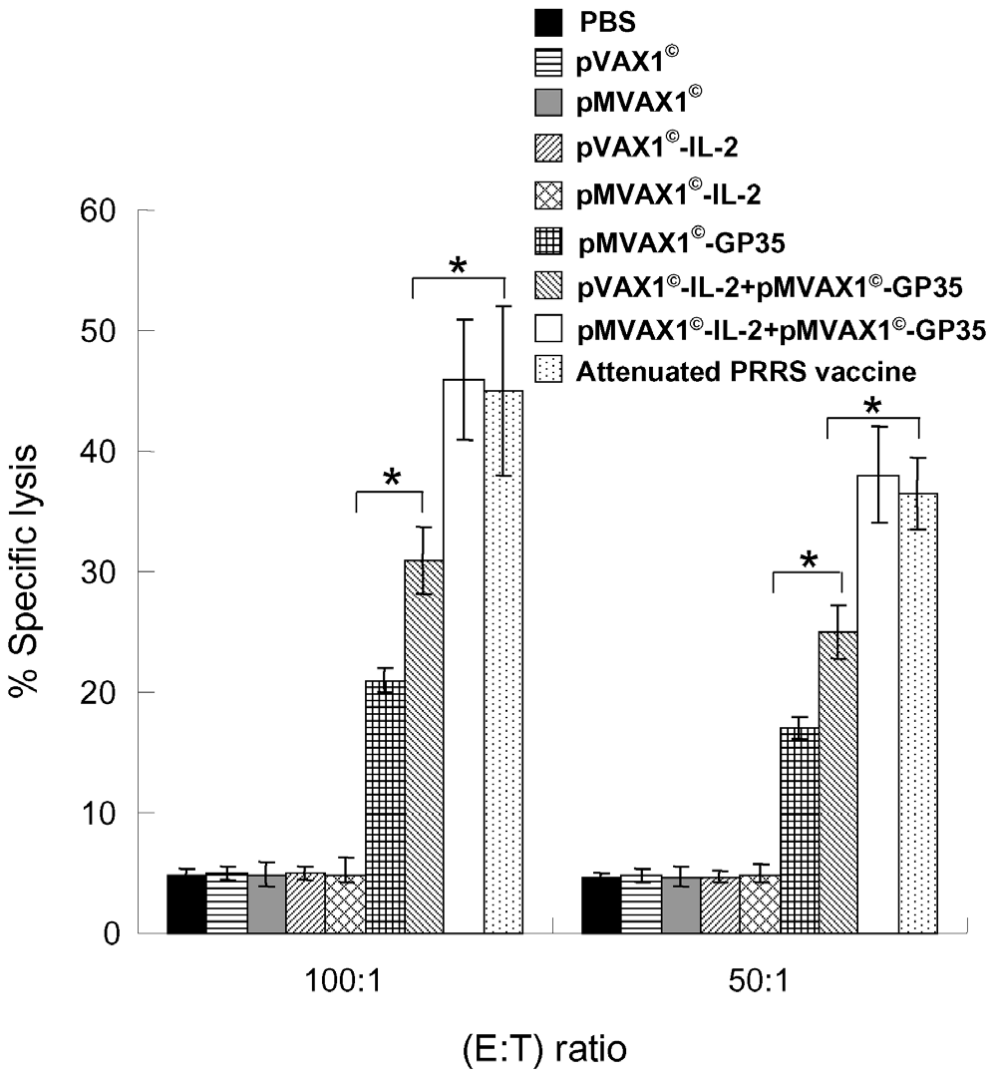
CTL responses in pigs immunized with PBS, individual plasmid or attenuated PRRS vaccine. PBMCs isolated from pigs (n = 5) at 42 dpi were used as effector cells. PAMs infected with PRRSV SD-JN were used as the target cells. The assays were performed in triplicate at effector cell/target cell (E:T) ratios of 100∶1 and 50∶1. After 4–6 h of incubation, the supernatant was harvested, and the substrate tetrazolium salt was added. The OD values were determined at 490 nm in an ELISA reader. Data were shown as mean±standard error.

CTL responses were determined using a LDH release assay against PRRSV infected PAMs. PBMCs isolated from pigs were incubated with target cells at different E:T ratios. The results were shown in Fig. 8. Compared to pVAX1^©^-IL-2+pMVAX1^©^-GP35 group, enhanced PRRSV-specific CTL responses were observed in the groups of pMVAX1^©^-IL-2+ pMVAX1^©^-GP35 and attenuated PRRS vaccine (*P*<0.05). In addition, the CTL activity in the pVAX1^©^-IL-2+pMVAX1^©^-GP35 immunized group was stronger than the pMVAX1^©^-GP35 group (*P*<0.05).

### Body temperature change and clinical signs after challenge

**Figure 9:**
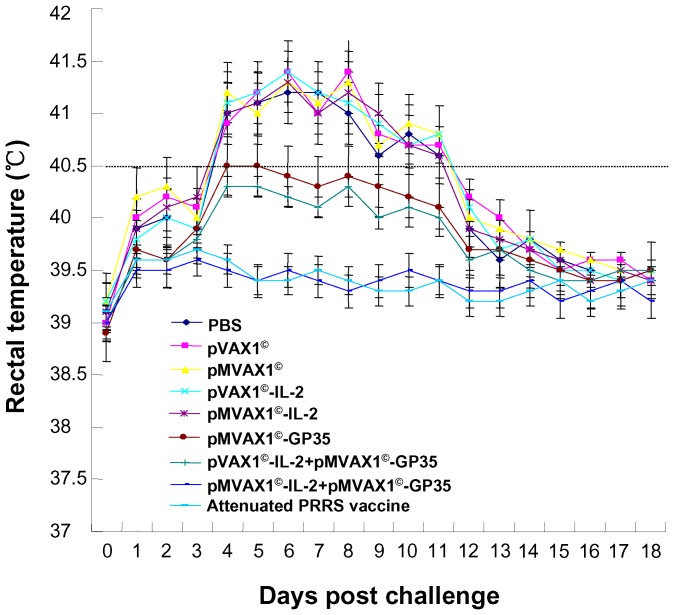
Rectal temperature of the pigs after challenge with HP-PRRSV strain SD-JN. The values were expressed as mean±standard error.

After challenge with HP-PRRSV, all pigs in PBS, pVAX1^©^, pMVAX1^©^, pVAX1^©^-IL-2, and pMVAX1^©^-IL-2 control groups had elevated body temperature (≥40.5°C) and displayed a range of clinical signs, including inappetence, lethargy, rough hair coats, skin cyanopathy, dyspnoea, coughing and shivering. Compared to the control groups, very low fever, very little fluctuation of rectal temperatures and very light clinical signs were observed in the group of pVAX1^©^-IL-2+pMVAX1^©^-GP35 (Fig. 9, Table 1). The score of clinical signs and rectal temperatures of the pigs in pMVAX1^©^-GP35 group was between pVAX1^©^-IL-2+pMVAX1^©^-GP35 and control groups. Importantly, pigs vaccinated with pMVAX1^©^-IL-2+pMVAX1^©^-GP35 and attenuated PRRS vaccine almost maintained normal body temperature (≤39.5°C) and showed no clinical signs during the 21 dpc (Fig. 9, Table 1).

**Table 1:** The scores of clinical signs of the pigs after challenge and lung lesions were recorded at 21 dpc.

Groups	Clinical signs scores b	Lung lesions scores c
PBS	10.1±0.46 A	85.7±7.22 A
pVAX1^©^	9.8±0.81 A	84.3±8.39 A
pMVAX1^©^	9.6±0.92 A	85.9±8.23 A
pVAX1^©^-IL-2	9.7±0.94 A	86.3±8.55 A
pMVAX1^©^-IL-2	9.5±0.79 A	83.2±7.64 A
pMVAX1^©^-GP35	6.1±1.02 B	41.2±9.33 B
pVAX1^©^-IL-2+pMVAX1^©^-GP35	4.2±0.56 C	19.3±4.51 C
pMVAX1^©^-IL-2+pMVAX1^©^-GP35	3.0±0.00 D	2.3±1.78 D
Attenuated PRRS vaccine	3.0±0.00 D	3.1±1.96 D

Lung lesions were also recorded when pigs died during the 21 days post challenge ^a^.

a Within each column, values followed by different letters (A, B, C and D) were significantly different (*P*<0.05).

b Clinical signs of each pig were evaluated daily after challenge. The results were expressed as mean±standard error.

c Average lung scores were recorded. The results were expressed as mean±standard error.

### Viremia after challenge

At 0, 7, 14 and 21 dpc, the blood samples of the pigs were collected and presence of PRRSV in the sera was determined by the appearance of CPE in MARC-145 cells. As shown in Table 2, 1 pig in attenuated PRRS vaccine group was PRRSV-positive at the time of challenge. Following challenge, viremia could not be detected in pigs vaccinated with pMVAX1^©^-IL-2+pMVAX1^©^-GP35 and attenuated PRRS vaccine, 3 of 5 pigs in pMVAX1^©^-GP35 group and 2 of 5 pigs in pVAX1^©^-IL-2+pMVAX1^©^-GP35 group were viremic at 14 dpc. In contrast, virus still could be recovered from pigs in PBS, pVAX1^©^, pMVAX1^©^, pVAX1^©^-IL-2 and pMVAX1^©^-IL-2 groups at 21 dpc (Table 2).

**Table 2:** Development of viremia in vaccinated animals.

Groups	Days post challenge (dpc)			
	0	7	14	21
PBS	0/5 a	5/5	3/3	1/1 b
pVAX1^©^	0/5	5/5	2/2	1/1
pMVAX1^©^	0/5	5/5	3/3	1/1
pVAX1^©^-IL-2	0/5	5/5	3/3	1/1
pMVAX1^©^-IL-2	0/5	5/5	2/2	1/1
pMVAX1^©^-GP35	0/5	4/5	3/5	0/5
pVAX1^©^-IL-2+pMVAX1^©^-GP35	0/5	3/5	2/5	0/5
pMVAX1^©^-IL-2+pMVAX1^©^-GP35	0/5	0/5	0/5	0/5
Attenuated PRRS vaccine	1/5	0/5	0/5	0/5

a Serum samples were collected weekly post challenge and virus isolation was performed by MARC-145 cell inoculations.

b The value in the denominator was the number of live pigs at that time point after challenge.

### Pathological examination

At 21 dpc, all live pigs were euthanized and the scores of lung lesions were evaluated. Lung lesions were also evaluated when pigs died during the 21 days post challenge. The results showed that all pigs from PBS, pVAX1^©^, pMVAX1^©^, pVAX1^©^-IL-2 and pMVAX1^©^-IL-2 control groups had diffuse tan consolidation of the lungs. Compared to these control groups, pigs inoculated with pVAX1^©^-IL-2+pMVAX1^©^-GP35 showed significantly lighter lung lesions (*P*<0.001). The score of lung lesions of pMVAX1^©^-GP35 vaccinated group was between pVAX1^©^-IL-2+pMVAX1^©^-GP35 and control groups (Table 1). However, lung lesions could be hardly observed in pigs vaccinated with pMVAX1^©^-IL-2+pMVAX1^©^-GP35 and attenuated PRRS vaccine, comparing to pVAX1^©^-IL-2+pMVAX1^©^-GP35 group (*P*<0.05, Table 1).

**Figure 10:**
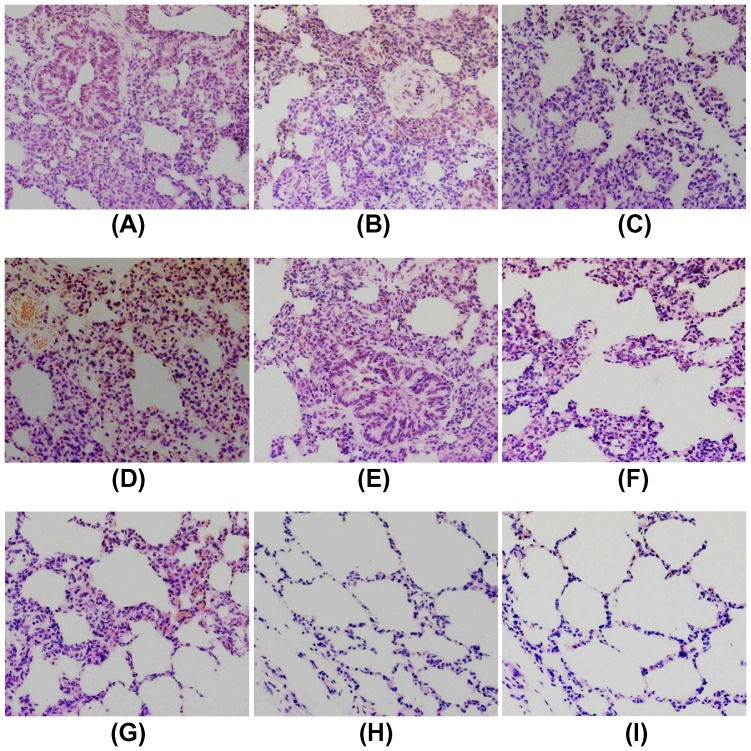
Examination of histological lesions in lungs of the pigs in PBS (A), pVAX1^©^ (B), pMVAX1^©^ (C), pVAX1^©^-IL-2 (D), pMVAX1^©^-IL-2 (E), pMVAX1^©^-GP35 (F), pVAX1^©^-IL-2+pMVAX1^©^-GP35 (G), pMVAX1^©^-IL-2+pMVAX1^©^-GP35 (H) and attenuated PRRS vaccine group (I) at 21 dpc. Hematoxylin and eosin staining (HE). Magnification, 200×.

On histological examination, lungs lesions of the pigs in PBS, pVAX1^©^, pMVAX1^©^, pVAX1^©^-IL-2 and pMVAX1^©^-IL-2 control groups were characterized by thickened alveolar walls, infiltration with intensive macrophage cells, and increased amounts of bronchiole exudates. However, the interstitial pneumonitis was hardly observed in pMVAX1^©^-IL-2+pMVAX1^©^-GP35 and attenuated PRRS vaccine groups, which was significantly milder than that in pVAX1^©^-IL-2+pMVAX1^©^-GP35 and pMVAX1^©^-GP35 groups (Fig. 10).

## Discussion

PRRSV infection is a global problem in swine industry. Recently, PRRS genetic engineering vaccines have been reported, including mycobacterium bovis BCG, pseudorabies virus or DNA vaccine expressing GP5 and M [18], [19], [33], recombinant fowlpox virus, adenovirus or DNA vaccine co-expressing GP3 and GP5 [6]–[10]. In order to increase the efficiency of the vaccine, an alternative approach is to co-deliver cytokines to enhance the immune response of PRRSV, including IL-18 [7], HSP70 [8], GM-CSF [9] and IFNα/IFNγ [10]. However, these PRRS genetic engineering vaccines cannot induce sufficient protective immunity against PRRSV challenge. In this study, porcine IL-2 and GP3-GP5 of PRRSV were expressed by the gene regulatory plasmid pMVAX1^©^. It was found that pMVAX1^©^-GP35 could express higher level of GP3-GP5 of PRRSV *in vitro*, induce significantly higher PRRSV-specific humoral immune responses and cell mediated immune responses in mice than pVAX1^©^-GP35. In addition, pMVAX1^©^-IL-2 could significantly enhance humoral immune responses, T lymphocyte proliferation responses, Th1-type and Th2-type cytokine responses and CTL responses of pMVAX1^©^-GP35 in pigs and pMVAX1^©^-IL-2+pMVAX1^©^-GP35 could provide complete protection against homologous HP-PRRSV challenge, similar with attenuated PRRS vaccine.

It was reported that insertion of the rabbit β-Globin Intron II gene sequence upstream of the F coding region of RSV could circumvent the tendency of RSV F RNA to undergo aberrant splicing and increase the protein expression *in vitro* [22]. Here a modification of pVAX1^©^ vector by including the rabbit β-Globin Intron II gene sequence under the control of a CMV promoter results in higher levels of GP35 or IL-2 expression *in vitro*. The tendency of GP35 or IL-2 RNA to undergo aberrant splicing might be circumvented. As a result, the expression level and immunogenicity of GP35 or IL-2 were improved.

It was reported that GP3 of PRRSV can induce neutralizing antibody and plays an important role in clearing the virus infection [34]. GP5 can induce higher titers of neutralizing antibody, and three B-cell epitopes in GP5 have been identified using monoclonal antibodies [35], [36]. The results of SN assays showed that pMVAX1^©^-GP35 could induce higher neutralizing antibodies than pVAX1^©^-GP35 in mice (Fig. 3B). In addition, PRRSV-specific T lymphocyte proliferation responses from the group of pMVAX1^©^-GP35 were significantly higher than the group that received pVAX1^©^-GP35 in mice (Fig. 4). So pMVAX1^©^-GP35 was selected as PRRS DNA vaccine candidate and further studied in pigs. pVAX1^©^-GP35 cannot provide efficient protection in pigs in our previous report [10] and was not investigated any further. PRRSV-specific humoral immune response, cell mediated immune response and protection provided by pMVAX1^©^-GP35 might be better than pVAX1^©^-GP35 in pigs, but it needs further studies and comparisons in the future.

It was found that pigs vaccinated with pMVAX1^©^-IL-2+pMVAX1^©^-GP35 had similar neutralizing antibodies, T lymphocyte proliferation responses, Th1-type cytokine IFN-γ and CTL responses with attenuated PRRS vaccine. It might be related to the highly efficient expression of IL-2 and GP35 by the gene regulatory plasmid pMVAX1^©^. However, the level of Th2-type cytokine IL-4 from the group of attenuated PRRS vaccine was significantly lower than that from pMVAX1^©^-IL-2+pMVAX1^©^-GP35 group, indicating attenuated PRRS vaccine mainly potentiates Th1-type cytokine responses while IL-2 facilitates both Th1-type and Th2-type cytokine responses.

To investigate the level of protection elicited by pMVAX1^©^-IL-2+pMVAX1^©^-GP35, the pigs were challenged with HP-PRRSV strain SD-JN. Temperature, clinical signs, lung lesions, viremia and histological pathology of lungs were examined to evaluate the protective efficiency. The results showed that all pigs in PBS, pVAX1^©^, pMVAX1^©^, pVAX1^©^-IL-2 and pMVAX1^©^-IL-2 control groups had high fever, showed severe clinical signs and lung lesions. However, pigs inoculated with pMVAX1^©^-IL-2+pMVAX1^©^-GP35 and attenuated PRRS vaccine had normal body temperature, showed no clinical signs and minimal lung lesions (Fig. 9, Table 1), which was coincident with the viraemia and histological examination (Fig. 10, Table 2). The enhanced PRRSV-specific humoral and cell mediated immunity induced by pMVAX1^©^-IL-2+pMVAX1^©^-GP35 could provide complete protection against homogenous challenge.

In summary, pMVAX1^©^-IL-2+pMVAX1^©^-GP35 could provide similar protective efficiency with attenuated PRRS vaccine. To our knowledge, this study was the first demonstration that porcine IL-2 and GP3-GP5 of PRRSV were expressed under the control of rabbit β-globin intron II gene and pMVAX1^©^-IL-2 could significantly enhance the humoral immune responses, cell mediated immune responses and CTL responses of pMVAX1^©^-GP35. Co-administration of pMVAX1^©^-IL-2 and pMVAX1^©^-GP35 might be attractive candidate vaccines for preventing HP-PRRSV infection.

## Supporting Information

Table S1 The ARRIVE Guidelines Checklist-NC3Rs for Animal Research.
(DOC)
